# Grand Challenge Veterinary Imaging: Technology, Science, and Communication

**DOI:** 10.3389/fvets.2015.00038

**Published:** 2015-09-30

**Authors:** Fintan J. McEvoy

**Affiliations:** ^1^Department of Veterinary Clinical and Animal Sciences, University of Copenhagen, Copenhagen, Denmark

**Keywords:** veterinary imaging, teleradiology, translational models, clinical veterinary radiology, machine learning

## 1 Introduction

Veterinary radiology is a long established subject discipline in veterinary science. It is fair to say that every veterinary graduate alive today will have received formal training in this subject irrespective of their date or place of graduation. Despite or perhaps because of this long ancestry, it is worthwhile examining the extent of the subject’s boundaries and its place in veterinary medicine. One can ask what imaging modalities fall under the remit of the subject and why they do so. Veterinary activities have always been concerned with the diagnosis and treatment of disease, with clinical and experimental animal research, and with agriculture, to select but a few areas of interest. How great a contribution can veterinary imaging make to these areas? The limits of what radiologists can and cannot do becomes unclear as the breakdown of traditional barriers between disciplines, considered essential to progress in medical education and scientific progress, continues (1). It has always been the case that the collection and use of image-related data are not the sole preserve of veterinary radiologists; uncertainties can exist as to who should be involved. The radiology community, both users and suppliers alike, has to ask what value imaging brings to collaborative work and how this value is best realized (2).

If uncertainties are accepted, they can be seen to arise from a number of recognizable factors. Some are longstanding while others are quite new. Important factors relate to the wide range of veterinary involvement in biological sciences and the roles that veterinarians take on in society. As the profession’s sphere of involvement extends, so too do the frontiers that veterinary imaging reach. Also long a factor, but certainly an accelerating trend, is the arrival of new technologies, such as multi-slice imaging that generates data in quantities, orders of magnitude greater than their predecessors. A newer phenomenon is the rise of widely available Patient Archive and Communications Systems (PACS). These can separate the radiologist from the clinic, or to put it more positively, can allow non-centralized clinicians access to radiologists at central remote sites. Having to deal with new technologies has always been a feature of radiology. This is seen from its far past with the transition from saving images on glass plates to the use of emulsions mounted on celluloid, from manual to automated film processing and more recently, from two-dimensional imaging to three-dimensional multislice technologies that allow volumetric acquisitions. The last of these, which allows sub-millimeter image slices, have the same dimensions in three orthogonal planes. These isovolume voxels represent a massive amount of data and allow superior planar reconstructions which in turn demand new approaches to reading studies (3). Each transition represents an order of magnitude change and in each case the radiology imaging community has risen to the challenge.

This article will trace some of these issues in the hope of seeing how they might be extrapolated into the future. We are all radiologists some of our working time and a very few of us are radiologists all of the time. It will benefit each individual along this spectrum of involvement, to have an awareness of each other’s presence. By looking at past changes and future prospects, we can better appreciate the subject that is veterinary imaging in all its manifestations.

## 2 Imaging Modalities

**Figure 1:**
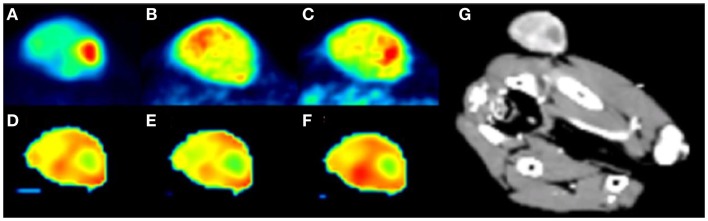
**PET and dynamic contrast enhanced perfusion CT (DCE-pCT) maps from a subcutaneous hemangiopericytoma in the gluteal region of a dog**. The PET tracers used included FDG, which is correlated to expression levels of glucose transporter proteins and hexokinases in cancer cells, to the functionality of regional microvasculature and to proliferative activity. The other tracer used was 64Cu-ATSM. It has an incompletely understood trapping mechanism. **(A)** FDG PET, **(B)** 3 h 64Cu-ATSM PET, **(C)** 24 h 64Cu-ATSM PET, **(D)** DCE-pCT blood flow, **(E)** DCE-pCT blood volume, **(F)** DCE-pCT permeability, and **(G)** maximum intensity projection. All images are in individually optimized window levels. The study was concerned with measuring tumor hypoxia and looked at the comparative performance of FDG and ATSM as hypoxia markers. Nine dogs were included and it was shown that in general a strong correlation exists between the ratio of maximum tumor uptake of 64Cu-ATSM to mean 64Cu-ATSM in muscle at 24 h and the maximum standard uptake value (SUV) of FDG. Differences in uptake between the tracers were seen in hypo-perfused areas. The dog shown here had a hypo-perfused region which showed the highest tumor FDG SUV, moderate 64Cu-ATSM uptake at 3 h and strong uptake at 24 h. It was concluded that the two tracers provide different biological information with overlapping spatial distribution in canine tumors, and that these differences were possibly related to tumor perfusion [based on Ref. (5)].

Veterinary imaging has the privilege and challenges that go with continued development of current and new imaging technologies and modalities. Despite the range of imaging technologies, most of them are based on either sound or electromagnetic waves or a combination thereof. They provide both anatomical and functional information; the two are to some extent mutually exclusive, so hybrid techniques or novel imaging agents are used to bring functionality to anatomical studies and *vice versa*. Thus, positron emission tomography (PET) images are superimposed upon MRI and CT images (Figure 1), and intravenous contrast is employed in CT and ultrasound imaging. Optical imaging in an emerging modality that promises information on morphology, physiology, and tissue composition (4). Simplifying our modalities to tissue interactions with sound or electromagnetic waves is useful for the purposes of overview but in their application, even the modalities that fall under the remit of the veterinary radiologist, digital radiography – computed radiography, magnetic resonance imaging, scintigraphy (including PET in limited cases) and ultrasound imaging, are so complex and nuanced in their use that few individuals can be completely at ease with all of them. Some degree of specialization will naturally result, raising questions about the need for certification and at what stage in training it should occur. Uncertainty remains as to level of specialization the market will bear and also whether complexity in modalities will continue to increase.

## 3 Provision of Service

Adding value to the management of clinical cases or to research involving animals is the goal of veterinary imaging. Value is difficult to define and there are few studies that objectively quantify the value of veterinary imaging. Outcomes of value such as the reduction of pain and suffering in animals, the provision of imaging reports that impact directly on case management and the collection of image data that can be used to address a scientific hypothesis all come to mind. Such outcomes may seem self evident to many readers, but pet insurance companies, corporate owners of veterinary practices, and funding bodies for research demand objective data such that being self evident may not be sufficient. Studies that examine the added value of imaging interventions will be increasingly welcome. It is important that veterinary radiologists, and the clinicians and researchers they serve, have a part in seeing how value is defined and measured.

Teleradiology is having and will increasingly have a profound effect on the supply and form of veterinary imaging services. Good radiography and good radiology can now be split into separate activities. Smaller clinical units that can obtain high quality radiographs can access high quality image interpretation. Also, establishments with very sophisticated equipment now have the freedom to dispense with full time on site radiologist services, preferring to choose the services of one or more distant radiologists. A positive feedback mechanism in the growth of teleradiology may be at play. Twenty to 30 years ago, few individual imaging studies were read by a radiologist. As ease of image acquisition and digital transfer increases, and teleradiology services reduce turnaround time, referral to teleradiology becomes more attractive clinically and economically for the practice. Use of teleradiology services in turn becomes increasingly the standard of care. The quality of image interpretation will be limited to some extent by the quality of communication between the referring veterinarian and the provider of specialist veterinary imaging interpretations. Optimal outcomes require that both parties, the referring clinician and the veterinary radiologist, while separated geographically, become closer to each other in mind set. The radiologist might be seen more as a specialist clinician, involved in selecting initial and follow up imaging studies and providing feedback on early image evaluations performed by the clinical team in addition to the more conventional image interpretation. If there is such a meeting of minds, the radiologist can provide clinically relevant information tailored to a clinical scenario that is also clear and well defined. The clinician’s skills in clinical history taking, physical examination and problem formulation are needed now perhaps more than ever before. If these challenges are met, then patient care will benefit; if unmet, clinical questions will be vague, image reports non-specific, and patient care will decline.

The increased utilization of hospital PACS and radiology information systems (RIS) will likely bring changes even within hospitals. The improved interaction between the radiologist and the case record will pull the radiologist into closer involvement with the patient. PACS and RIS systems are just a small part of the field of Radiology Informatics. It is a field that will impact on every aspect of veterinary radiology, from training to the provision of service and research (6).

## 4 Training

Attracting dedicated individuals and providing training and certifying expertise are important, perhaps even the dominant activities of the various specialty colleges such as the American College of Veterinary Radiology and the European College of Veterinary Diagnostic Imaging. Both Colleges comprise large numbers of highly skilled Diplomates but it would of course be wrong to think that they are the sole repositories of such skills. Though not a certifying body, the International Veterinary Radiology Association by virtue of its members and geographical spread is perhaps best positioned to gain a complete view of specialization throughout the world. It is helpful for colleges and other bodies certifying specialist skills to communicate, so that even allowing for regional differences, common purpose is sought and common needs are met. The aims of specialty colleges are clear and well established. Continuing professional development courses are other important sources of further training in veterinary imaging. Here, the goal may be less well defined, content may range from a simple update intended to reawaken interest on a topic to a detailed review of one specific aspect of a subject. Training outcomes may or may not be tested and certified.

Training in all its forms and at all levels in imaging is to be encouraged since involvement with the subject is so wide. Participants will be most content when the extent of that training is precisely defined. Also, awareness of overlapping areas such as surgery and medicine is important for the radiologist to contribute fully. The public who seek specialist services also require clear guidance on the training qualification of the imaging providers they encounter. This is easier said than done, since continuing education providers may wish to make great claims for the outcome of their courses. It is important that these providers make attempts to benchmark their courses, seeking assistance from universities, national veterinary bodies and established colleges. The challenge is to encourage the provision of training opportunities and at the same time require the providers to document learning outcomes.

Up to date and active specialists are needed. Training and retaining these individuals in veterinary imaging is not trivial; the challenge in doing so, and the consequences of failure are well recognized (7).

## 5 Research and Development

There is a great attraction in applying medical imaging methodologies to veterinary patients. Spontaneous disease in our patients is often offered and usually well received as a spontaneous model for disease in humans (8). The size of lesions in our common patients, dogs and cats can be similar to that encountered in humans. Such lesions are thus suited to the spatial resolutions available in medical imaging equipment. It is very important that the veterinary imaging community is fully involved in such projects. Collaborating with researchers investigating disease in humans can give us access to front line procedures in imaging at the forefront of imaging technology, for example, in the investigation of novel PET tracers. In this way, the individual patient involved can benefit by having access to a widened range of diagnostic and treatment options, and in some cases veterinary science in general benefits from the insights gained in the collaboration. This research, however, is only part of our story and we must remember that the technologies and procedures used, may or may not in the foreseeable future be transferable to veterinary patients. Research that on the face of it is less high profile, employing less advanced technology can nonetheless be leading edge and provide valuable diagnostic information for the prognosis and treatment of our veterinary patients. The value of a research result is less a function of the sophistication of the imaging tool employed than of the actual research hypothesis being tested. There must be a place for veterinary research projects that address the needs of veterinary patients and satisfy our own curiosity about the biological systems that surround us.

## 6 Technical Advances

**Figure 2:**
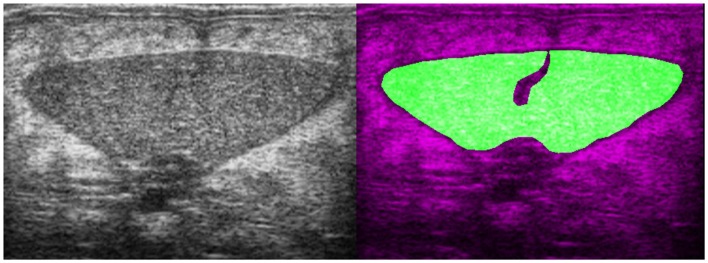
**Transverse B-mode ultrasound image and image mask from a European eel (*Anguilla anguilla*)**. The mask (right image) shows the area of the ovaries that have been artificially induced to maturation. Machine learning algorithms use texture parameters to classify ovarian maturation in these fish. This work is part of the PRO-EEL project (www.pro-eel.eu) and received funding from the 7th Framework Programme of the European Commission under the theme “Food, Agriculture and Fisheries, and Biotechnology”.

Technical advances have had an enormous impact on veterinary imaging in the lifetimes of contemporary radiologists. The wide availability of ultrasound and increasing presence of computed tomography and magnetic resonance imaging are developments that have been rapid and perhaps unforeseen a few decades ago. These are the products of engineering, and to some extent the medical community has been a peripheral beneficiary of developments in other fields. Engineering, computer science, and molecular biology are three principal disciplines that over recent decades have impacted on the medical imaging field and resulted in such astounding progress. Innovation will continue, and medical imaging as well as veterinary imaging will benefit. What those innovations will be and how they will impact the discipline is of course uncertain. One likely possibility is the increased application of machine learning in medical imaging. Automated hand-written character recognition, face recognition in our cloud photograph albums, and context relevant information displayed on our smart phones are current realities. These powerful, value adding applications of machine learning could transfer to our domain (Figure 2). Machine learning algorithms require access to massive amounts of well classified data in order to learn. With better integration of hospital information systems, this is technically possible but difficult for medico-legal reasons as patient data need to be shared. Veterinary imaging is better positioned than medical imaging with regard to such sharing as the regulations are less problematic. Successful application of pattern recognition software to medical images for use as a radiologist’s assistant cannot be too distant a prospect (9, 10). And then there may be some surprises: outliers, developments initially far removed from medicine, that suddenly cross over and have major impact. Graphene for example is a new material that is quickly finding use in multiple spheres (11). It may bring us low resistance conductors or superconductors that could reduce costs and thus increase the availability of magnetic resonance imaging. If this were to happen, MRI in 20 years may be as common as ultrasound is today. The advance may not come from this particular material but it is likely there are innovations to come that will bring opportunities and challenges for the next generation. Veterinary researchers have been and should continue to be, open to new developments as they arise. Working at the edge of one’s domain is often useful, and in imaging the veterinary radiologist is best positioned to see potential clinical benefit in a novel technology.

## 7 Conclusion

The various challenges facing the veterinary imaging community are more exciting than problematic. If there is a problem, it will be to assemble and coordinate as many skilled minds, from as many spheres of activity as possible, to focus on advancing our field. A part of this will be to encourage that wide community to publish and share their hypothesis and findings. Frontiers in Veterinary Imaging is one avenue for this process.

## Conflict of Interest Statement

The author declares that the research was conducted in the absence of any commercial or financial relationships that could be construed as a potential conflict of interest.
